# Comparison of Two Simplified Versions of the Gielis Equation for Describing the Shape of Bamboo Leaves

**DOI:** 10.3390/plants11223058

**Published:** 2022-11-11

**Authors:** Weihao Yao, Ülo Niinemets, Wenjing Yao, Johan Gielis, Julian Schrader, Kexin Yu, Peijian Shi

**Affiliations:** 1 Bamboo Research Institute, College of Biology and the Environment, Nanjing Forestry University, Nanjing 210037, China; 2 Institute of Agricultural and Environmental Sciences, Estonian University of Life Sciences, 51006 Tartu, Estonia; 3 Estonian Academy of Sciences, 10130 Tallinn, Estonia; 4 Department of Biosciences Engineering, University of Antwerp, B-2020 Antwerp, Belgium; 5 School of Natural Sciences, Macquarie University, Sydney, NSW 2109, Australia

**Keywords:** leaf shape, percent error, *Pleioblastus*, polar angle, polar radius

## Abstract

Bamboo is an important component in subtropical and tropical forest communities. The plant has characteristic long lanceolate leaves with parallel venation. Prior studies have shown that the leaf shapes of this plant group can be well described by a simplified version (referred to as SGE-1) of the Gielis equation, a polar coordinate equation extended from the superellipse equation. SGE-1 with only two model parameters is less complex than the original Gielis equation with six parameters. Previous studies have seldom tested whether other simplified versions of the Gielis equation are superior to SGE-1 in fitting empirical leaf shape data. In the present study, we compared a three-parameter Gielis equation (referred to as SGE-2) with the two-parameter SGE-1 using the leaf boundary coordinate data of six bamboo species within the same genus that have representative long lanceolate leaves, with >300 leaves for each species. We sampled 2000 data points at approximately equidistant locations on the boundary of each leaf, and estimated the parameters for the two models. The root–mean–square error (RMSE) between the observed and predicted radii from the polar point to data points on the boundary of each leaf was used as a measure of the model goodness of fit, and the mean percent error between the RMSEs from fitting SGE-1 and SGE-2 was used to examine whether the introduction of an additional parameter in SGE-1 remarkably improves the model’s fitting. We found that the RMSE value of SGE-2 was always smaller than that of SGE-1. The mean percent errors among the two models ranged from 7.5% to 20% across the six species. These results indicate that SGE-2 is superior to SGE-1 and should be used in fitting leaf shapes. We argue that the results of the current study can be potentially extended to other lanceolate leaf shapes.

## 1. Introduction

The subfamily Bambusoideae, which includes >1300 species covering 75 genera of Poaceae, are important components in many ecosystems, and are particularly abundant in subtropical and tropical areas [1]. As typical to Poaceae, leaves of all bamboo species have parallel venation, and most species have long lanceolate leaves. Lin et al. [2] reported that the leaf lamina width/length ratio (referred to as leaf width/length ratio for convenience hereinafter) ranged from 0.05 to 0.35 for 101 bamboo taxa, and the interspecific variation in leaf shape is mainly due to differences in the leaf width/length ratio. When the leaf width/length ratio is large, the leaf shape of some bamboo species (e.g., *Shibataea chinensis*) appears to be ovate. In fact, in bamboos, leaf width/length ratio provides an objective criterion to distinguish among lanceolate or linear leaves and ovate leaves [3]. Given the importance of leaf shape in the resource harvesting and evolution of plants, several indices (e.g., leaf width/length ratio, leaf dissection index, leaf roundness index, leaf ellipticalness index, and the fractal dimension of leaf boundary) were proposed to quantify the leaf shape geometrical characteristics, especially the tapering and curvature of a leaf’s boundary [2,4,5,6,7]. However, the number of studies that have developed explicit models that can quantitatively describe leaf boundaries of the Poaceae species is very limited [8,9,10,11].

It would be highly beneficial to have a “universal” parametric model that can describe all natural geometries, such as the diverse leaf shapes across different plant groups; such an ambition stems from the successful use of general models in other natural science fields, especially in physics, where general laws have been defined since the Renaissance [12]. However, the variations in natural geometries, especially asymmetry, handedness, and spirality, have far exceeded what we can imagine based on the extant physical and mathematical knowledge. It is difficult to find a universal parametric model to describe all morphological variations in leaves across different plant groups. Fortunately, it is still hoped to find one that can apply to some groups. Gielis [13] proposed a polar coordinate equation, referred to as the Gielis equation hereinafter, which can simulate many geometries found in nature, although its capacity to describe actual biological objects has been seldom tested. The Gielis equation is a generalization of the superellipse equation [14], while the latter is a generalization of the ellipse equation. In recent years, several studies have demonstrated the validity of the Gielis equation for describing and fitting many natural geometries (see Ref. [15] and the references therein). The first practical application of the Gielis equation was a description of leaf shapes of four bamboo species from the genus *Indocalamus* [9], followed by Ref. [11], in which the leaf shapes of an additional 42 bamboo species were demonstrated to follow this equation.

The original Gielis equation has six empirical parameters, and its mathematical expression in the polar coordinate system is as follows:

(1) $$r(\varphi )={\left({\left|\frac{1}{A}\cos\left(\frac{m}{4}\varphi \right)\right|}^{n_{2}}+{\left|\frac{1}{B}\sin\left(\frac{m}{4}\varphi \right)\right|}^{n_{3}}\right)}^{-\frac{1}{n_{1}}}$$

where *r* and φ are the polar radius and polar angle, respectively; *A*, *B*, *n*_1_, *n*_2_, and *n*_3_ are parameters to be fitted; *m* is a positive integer that determines the number of angles of the Gielis curve within [0, 2π]. This equation can be re-expressed as [16,17]:

(2) $$r(\varphi )=a{\left({\left|\cos\left(\frac{m}{4}\varphi \right)\right|}^{n_{2}}+{\left|\frac{1}{k}\sin\left(\frac{m}{4}\varphi \right)\right|}^{n_{3}}\right)}^{-\frac{1}{n_{1}}}$$

where $a=A^{n_{2}/n_{1}}$ and $k=B/A^{n_{2}/n_{3}}$. To decrease the model’s complexity and more effectively fit the empirical boundary data of bamboo leaves, Shi et al. [9] used a simplified version of Equation (2) by setting *m* = 1, *k* = 1 and *n*_2_ = *n*_3_ =1, which is referred to as SGE-1 hereinafter:

(3) $$r(\varphi )=a{\left(\left|\cos\left(\frac{\varphi }{4}\right)\right|+\left|\sin\left(\frac{\varphi }{4}\right)\right|\right)}^{-\frac{1}{n_{1}}}$$

The SGE-1 was confirmed to provide very good fits to empirical leaf boundary coordinate data for the studied 46 bamboo species (the coefficients of determination were all larger than 0.985) [9,11]. The model parameter *n*_1_ characterizes the elongation change (accompanied with the change in tapering and curvature) of leaf shape, and it was significantly different among species, but it varied in a narrow range, from 0.02 to 0.10 [9,11]. However, the question is whether additional modifications of the Gielis equation can result in a model that describes the leaf shapes of bamboo with better goodness of fit, while keeping the number of fitted parameters low. Previously, the following simplified version of the original Gielis equation with an additional parameter *n*_2_, which can render the equation to generate more diverse symmetrical geometries [18], was used and shown to perform similarly to SGE-1 in fitting the shapes of avian eggs [19]:

(4) $$r(\varphi )=a{\left({\left|\cos\left(\frac{\varphi }{4}\right)\right|}^{n_{2}}+{\left|\sin\left(\frac{\varphi }{4}\right)\right|}^{n_{2}}\right)}^{-\frac{1}{n_{1}}}$$

We refer to Equation (4) as SGE-2. When *m* is set to 5 instead of 2, this model version can describe the shapes of some sea stars, and the geometries of the outer rims of corolla tubes of *Vinca major* [17,20].

In the present work, we sampled 1996 leaves from six bamboo species from the genus *Pleioblastus*, and compared the predictions using SGE-1 and SGE-2 to test whether SGE-2 can improve the model prediction of bamboo leaf shapes. 

## 2. Materials and Methods

### 2.1. Plant Materials and Leaf Collection

**Figure 1:**
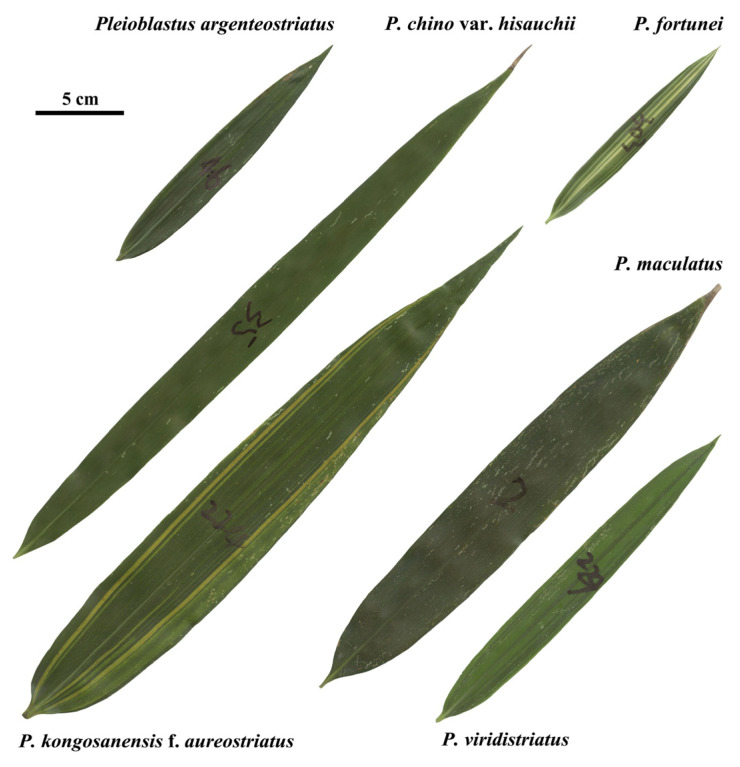
Outlines of leaf samples of six *Pleioblastus* species collected from the Nanjing Forestry University campus.

We sampled 1996 leaves of six *Pleioblastus* species (Figure 1 for the leaf samples) growing at the Nanjing Forestry University campus (118°48′35″ E, 32°4′67″ N) in late August 2021 when the leaves were fully mature in this season. For each species, we randomly sampled more than 300 leaves from different plant canopy positions without distinguishing among different canopy microenvironments and among leaf ages. For each species, leaves were sampled from 10 to 60 culms (Table 1 for sampling information). Although the accurate age of each culm cannot be determined, all species had been planted on this site more than 20 years ago. We argue that due to the large sample size, influences of sampling vertical positions, azimuth, leaf age, and culm age do not alter our results qualitatively. The leaves were wrapped in wet paper, and put into plastic self-sealing bags (45 cm × 34 cm) to reduce water loss. The bags with leaves were stored at 5 °C in a fridge for less than 24 h before scanning.

### 2.2. Data Acquisition

We scanned the fresh leaves with a photo scanner (V550, Epson, Batam, Indonesia) at 600 dpi resolution. The scanned color images were converted to black and white BMP files with Photoshop CS6, ver. 13.0 (Adobe, San Jose, CA, USA). Matlab (version ≥ 2009a; MathWorks, Natick, MA, USA) procedures developed by Refs. [10,21] were used to extract the planar coordinates of the boundary of each leaf. The boundary of each leaf was characterized by 2000 approximately equidistantly spaced coordinates using the “adjdata” function of the “biogeom” package (version 1.2.1) [15] in R (version 4.2.0) [22].

### 2.3. Data Fitting and Model Evaluation

We used two simplified versions of the Gielis equation, SGE-1 (Equation (3)) and SGE-2 (Equation (4)), to fit the boundary coordinates for each leaf using the “fitGE” function in the “biogeom” package (version 1.2.1) [15]. This function estimates the model parameters by minimizing the residual sum of squares (RSS) between the observed and predicted radii ($r_{i}$ vs. ${\hat{r}}_{i}$) from the polar point to the leaf boundary:

(5) $$\mathrm{RSS}=\sum_{i=1}^{N}{\left(r_{i}-{\hat{r}}_{i}\right)}^{2}$$

where *N* is the number of data points on the leaf’s boundary (*N* = 2000 in our study). The root–mean–square error (RMSE) was calculated to characterize the goodness of fit of the nonlinear regression:

(6) $$\mathrm{RMSE}=\sqrt{\mathrm{RSS}/N}$$

We used the paired *t*-test at the 0.05 significance level to compare the goodness of fits of the two models, SGE-1 and SGE-2. We further calculated the mean percent error (MPE) between the two groups of RMSEs:

(7) $$\mathrm{MPE}=\frac{1}{Q}\sum_{j=1}^{Q}\frac{{\mathrm{RMSE}}_{1,j}-{\mathrm{RMSE}}_{2,j}}{{\mathrm{RMSE}}_{1,j}}\times 100\%$$

where *j* represents the *j*-th leaf, and *Q* represents the number of leaves for each species. MPE was used to assess whether the introduction of an additional parameter in SGE-1 to form SGE-2 enhances model predictability enough to compensate for the increase in model complexity. As a rule of thumb, a >5% MPE indicates that it is worth adding an additional parameter [23].

For the estimated values of *n*_1_ and *n*_2_ in SGE-2, we used one-way ANOVA followed the Tukey’s HSD test [24] to examine whether the model parameters differed among any two species. Before comparing the parameter values among the species, the parameter values were log- or exp-transformed, depending on the shape of the parameter frequency distributions. For a right-skewed distribution (parameter *n*_1_), a logarithmic transformation was used; for a left-skewed distribution (parameter *n*_2_), an exponential transformation was used [25]. Estimated values of parameters and goodness of fit for models SGE-1 and SGE-2 for all the 1996 leaves are shown in Tables S1 and S2 in the online Supplementary Materials.

The statistical software R (version 4.2.0) [22] was used to carry out the statistical analyses and to draw figures. 

## 3. Results

**Figure 2:**
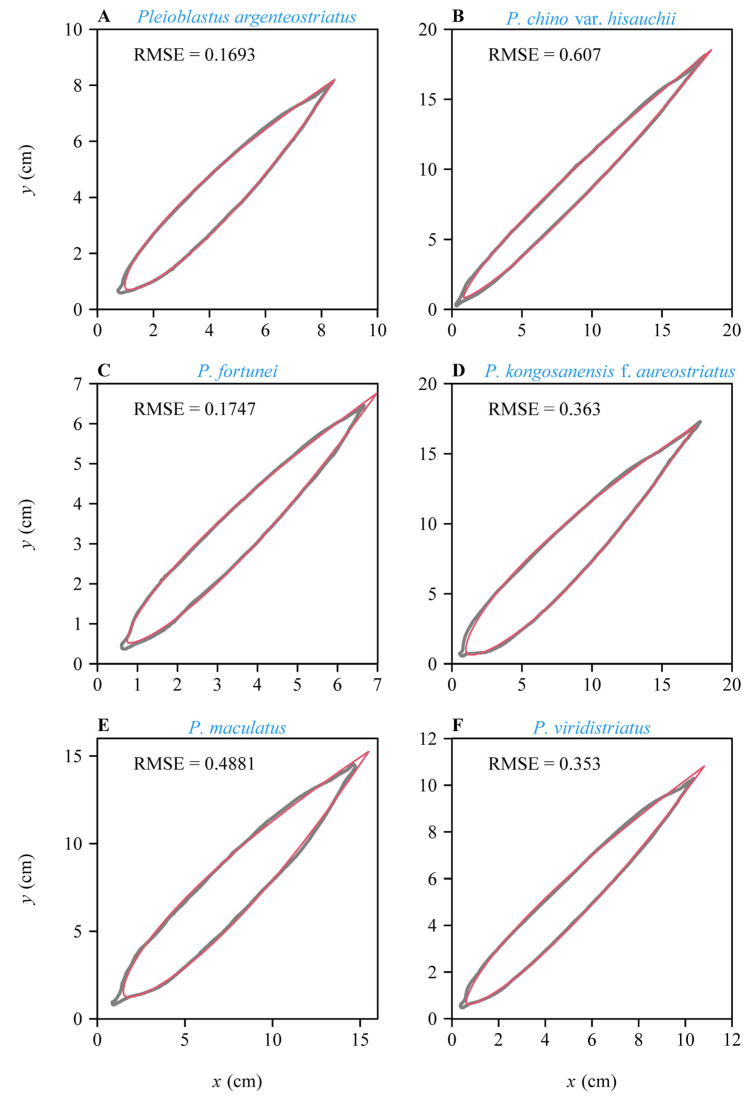
Illustration of the results of fitting the boundary coordinates of representative leaf samples for the six studied bamboo species (the same leaves as shown in Figure 1) using SGE-1. The gray curves are the actual scanned leaf boundaries; the red curves are the leaf boundaries predicted by the model SGE-1, i.e., Equation (3).

**Figure 3:**
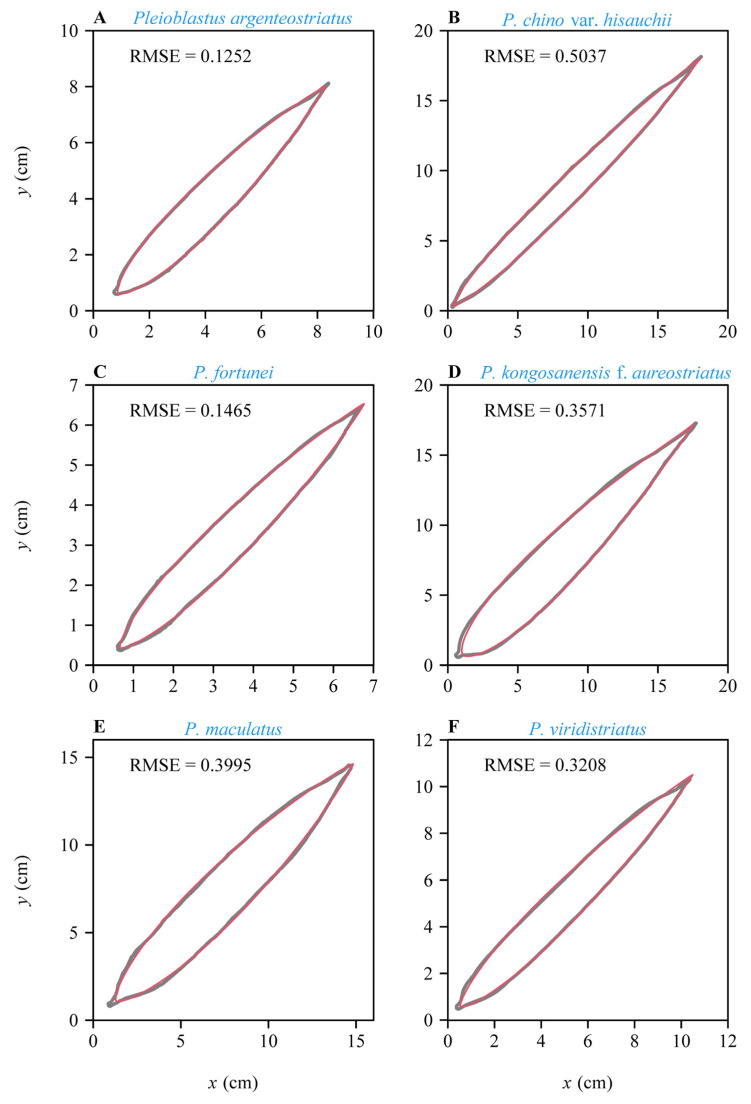
Illustration of the results of fitting the boundary coordinates of representative leaf samples for the six studied bamboo species (the same leaves as shown in Figure 1) using SGE-2. The gray curves are the actual scanned leaf boundaries; the red curves are the leaf boundaries predicted by the model SGE-2, i.e., Equation (4).

**Figure 4:**
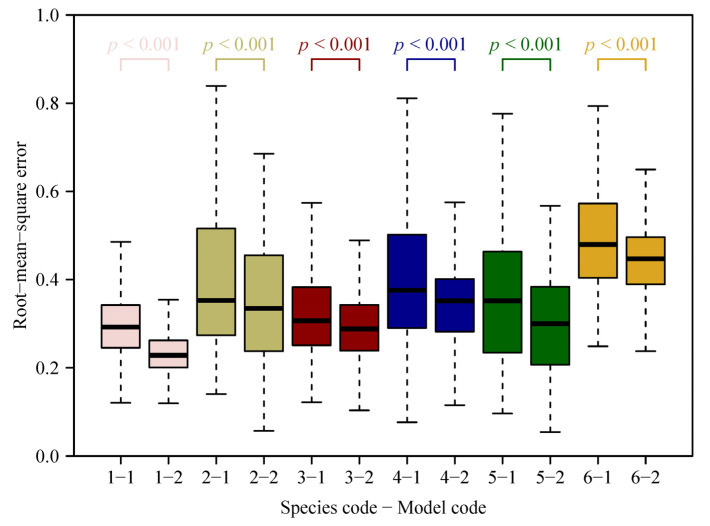
Comparison of the root–mean–square errors (RMSEs) between the two simplified Gielis models (SGE-1 and SGE-2, i.e., Equations (3) and (4)) for the studied six *Pleioblastus* species (Table 1 for species codes). The thick horizontal lines within the boxes represent median values of RMSEs; the box length represents the difference between the 3rd/4th quantile and the 1st/4th quantile; whiskers give 1.5 times the box length or maximum (or minimum) values. The two groups of RMSEs between the two models (1 and 2) for each species (1 to 6) were compared by a paired sample *t*-test.

**Figure 5:**
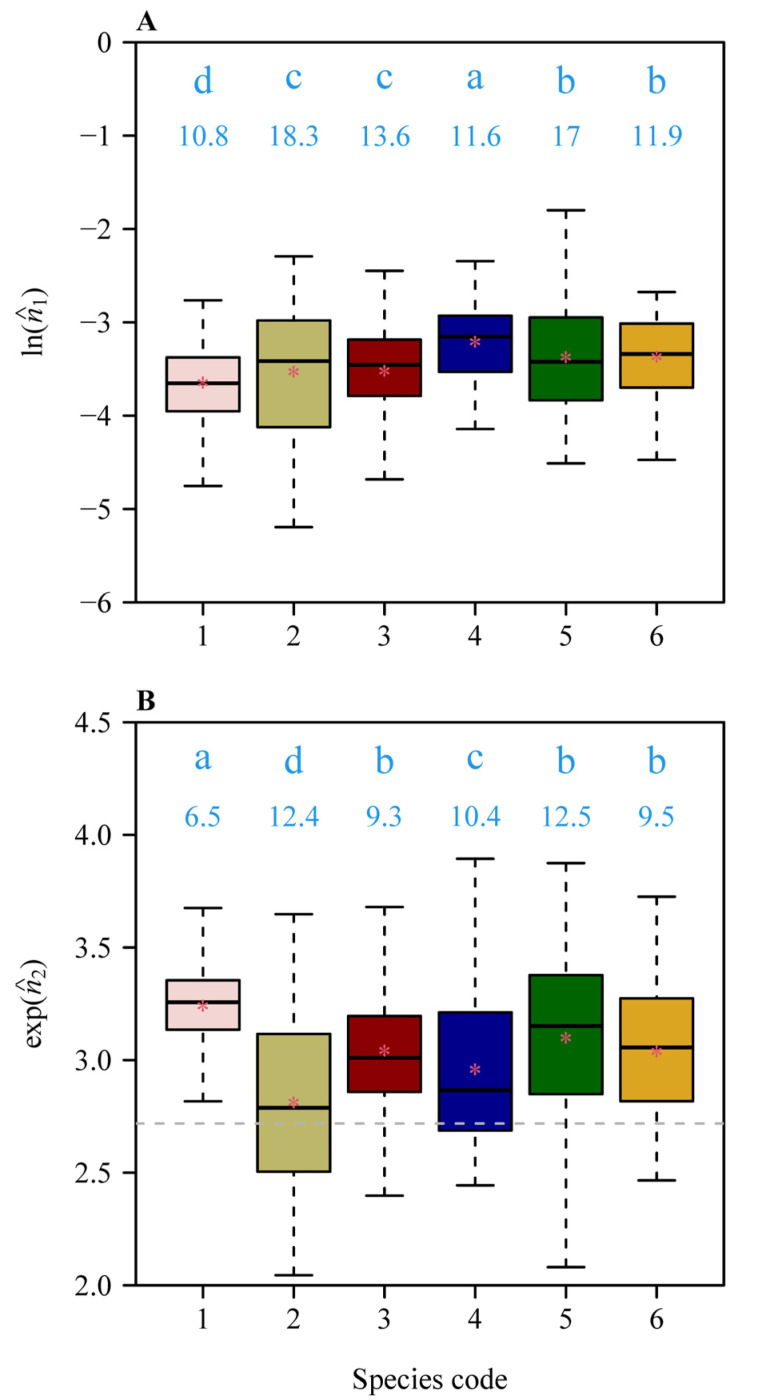
Comparisons of the log-transformed values of the model parameter *n*_1_ (**A**) and the exp-transformed values of the model parameter *n*_2_ (**B**) for the model SGE-2 (Equation (4)) for the six bamboo species (Table 1 for species codes). Different transformations reflect differences in the frequency distributions of the estimated parameter values (right-shewed for *n*_1_ and left-skewed for *n*_2_). In each panel, the lowercase letters show the significance of the differences in the estimated values between any two species at the 0.05 significance level. The numeric values at the top of each box provide the coefficients of variation (%). The horizontal solid line represents the median, and the red asterisk the mean. The whiskers provide the 1.5-fold interquartile range or maximum (or minimum) values. In (**B**), the horizontal gray dashed line shows exp(1).

Both models provided good fits to the boundary of leaves in all studies species (Tables S1 and S2 in the online Supplementary Materials; see Figure 2 and Figure 3 for the six leaf examples as intuitively shown in Figure 1). The RMSE varied among species, with the lowest RMSE observed for *Pleioblastus argenteostriatus* fitted with model SGE-2 and the highest RMSE observed for *P. viridistriatus* fitted with model SG-1 (Figure 4). Visually, the boundaries predicted by model SGE-2 more closely matched the actual leaf boundaries than those predicted by the model SGE-1 (Figure 3 versus Figure 2). This was confirmed by a comparison of the mean RMSEs among species. For all species, the RMSE for the model SGE-1 was greater than that for the model SGE-2 (all *p* values < 0.001; Figure 4). The mean percentage errors (MPEs) between the RMSEs for the two models (Equation (7)) were greater than 5% for all studied bamboo species (20.2%, 12.8%, 7.5%, 11.3%, 15.3%, and 8.5%, following species order in Table 1). That is, the introduction of *n*_2_ into SGE-2 largely improved the goodness of fit. The parameters, log(*n*_1_) or exp(*n*_2_), varied among the six species, reflecting differences in leaf elongation and margin curvature (Figure 5). All means of the estimated values of *n*_2_ of the six species were greater than 1, and most numerical values of *n*_2_ (1559 out of 1996) were greater than 1.0 (Figure 5B), further suggesting that an additional parameter needs to be incorporated.

## 4. Discussion and Conclusions

In the present study, we found that SEG-2 provided a better goodness of fit than SGE-1 in describing the shape of bamboo leaves. Shi et al. [19] found that SGE-2 also applies to the shape of avian eggs, but SGE-1 cannot reproduce the egg shapes of birds. In Equation (4), let us use an unknown parameter *m* to replace 1, i.e.,

(8) $$r(\varphi )=a{\left({\left|\cos\left(\frac{m}{4}\varphi \right)\right|}^{n_{2}}+{\left|\sin\left(\frac{m}{4}\varphi \right)\right|}^{n_{2}}\right)}^{-\frac{1}{n_{1}}}$$

Wang et al. [20] found that Equation (8) can describe the geometries of the outer rims of corolla tubes of *V. major* associated with the flowers that have five or four petals (where *m* = 5 and 4, respectively). Li et al. [26] found that Equation (8) is also applicable to the vertical projection’s shape (in the top view) of *Koelreuteria paniculata* fruit when setting *m* = 3. This equation has more applications to other natural geometries owing to its rich symmetrical characteristics (e.g., the profiles of some sea stars) [17,18]. SGE-1 can be regarded as a special case of SGE-2, where SGE-2 is a special case of Equation (8). It will be valuable in future to further examine the validity of Equation (8) for more biological specimens from the same taxon, but with a large variation in morphology (i.e., diatoms, cross-sections of some plant stems that exhibit apparent radial symmetry). 

**Figure 6:**
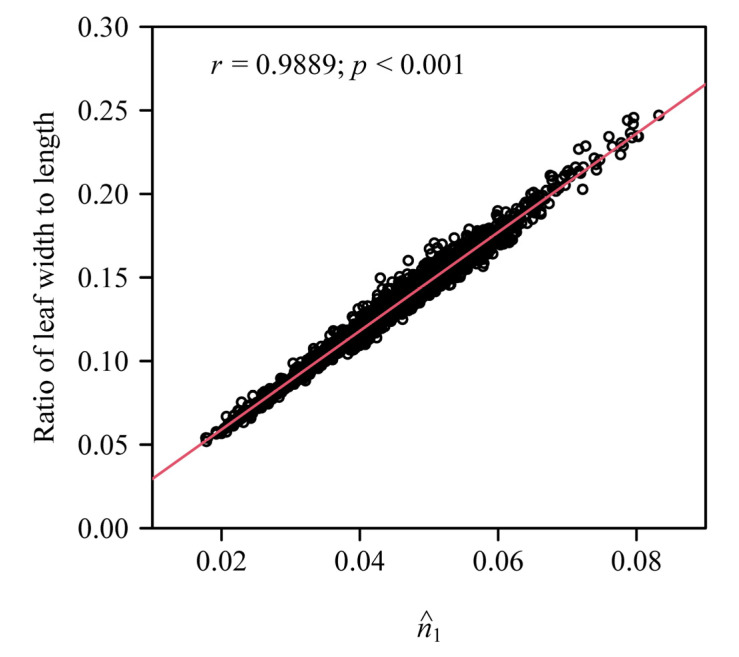
The correlation between the estimated values of the model parameter *n*_1_ for SGE-1 and the ratios of leaf width to length.

It is necessary to point out that SGE-1 only has two model parameters (*a*, as the leaf size parameter, and *n*_1_, as the leaf shape parameter), where *n*_1_ is positively correlated with the ratio of leaf width to length [10,21]. That is, *n*_1_ can be used in SGE-1 as a single leaf shape parameter to compare the differences in leaf shape across different bamboo species: a smaller *n*_1_ value corresponds to a narrower leaf with a sharper leaf base, while a greater *n*_1_ value corresponds to a broader leaf with a rounder leaf base [9,11]. Our data also confirm these results (Figure 6). However, there are two leaf shape parameters (i.e., *n*_1_ and *n*_2_) in SGE-2, which means it is not easy to explain the leaf shape variations within a species and across different species if we attempt to use *n*_1_ and *n*_2_ simultaneously for the quantification of leaf shape. In further analyses, we found that the leaf width/length ratio can be expressed as a function of *n*_1_ and *n*_2_ with a higher goodness of fit using the generalized additive models (e.g., models described in Refs. [27,28]). However, the interaction effect between the two parameters on data fitting is still difficult to explain (not shown due to the limitation of space). Thus, we suggest directly using the leaf width/length ratio to reflect or quantify the elongation change of leaf shape rather than using the two parameters in SGE-2. In fact, the leaf width/length ratio has been demonstrated to be closely correlated with the leaf fractal dimension [6]. The main role of SGE-2 in future research should not be to quantify the elongation change (accompanied with the change in tapering and curvature) of leaf shape, but it should be focused on the simulation of the intra- and interspecific variations in leaf shape based on the ranges of the two parameters’ empirical estimated values. Another strength of SGE-2 is its ability to simulate a lanceolate leaf whose leaf area can be accurately calculated based on the parameters, and it is valuable in studying the effects of leaf shape and size on leaf structural, chemical and physiological differentiation [29]. 

In the present study, we compared the validity of two simplified Gielis equations (SGE-1 with two model parameters and SGE-2 with three model parameters) using 1996 leaves from six bamboo species, with more than 300 leaves measured for each species. We found that SGE-2 better characterizes the shape for each of all studied bamboo species. Although SGE-2 is more complex from the viewpoint of the model’s structure than SGE-1, the mean percent errors for the six bamboo species were greater than 5%, which indicates that it is worthwhile to include an additional parameter in SGE-2 at the cost of increasing model complexity. Most numerical values of *n*_2_ (1559 out of 1996) were greater than 1.0, further suggesting that an additional parameter needs to be incorporated. This work provides a versatile model tool for the description of the leaf shape of bamboo and other plant species with similar lanceolate leaves.

## Figures and Tables

**Table 1:** Sampling information of the six bamboo species.

Species Code	Scientific Name	Number of Culms	Number of Leaves	Sampling Date
1	*Pleioblastus argenteostriatus*	60	335	27 August 2021
2	*Pleioblastus chino* var. *hisauchii*	15	336	21 August 2021
3	*Pleioblastus fortunei*	60	337	24 August 2021
4	*Pleioblastus kongosanensis* f. *aureostriatus*	60	336	22 August 2021
5	*Pleioblastus maculatus*	10	323	25 August 2021
6	*Pleioblastus viridistriatus*	60	329	23 August 2021

## Data Availability

The data used in the present work have been listed in the online Supplementary Materials.

## Supplementary Materials

The following supporting information can be downloaded at: https://www.mdpi.com/article/10.3390/plants11223058/s1, Table S1: The estimated values of parameters and goodness of fit using the SGE-1 to fit empirical leaf boundary data; Table S2: The estimated values of parameters and goodness of fit using the SGE-2 to fit empirical leaf boundary data.
